# A retrospective and in silico study on the augmentation of risperidone in the treatment of adolescent treatment-resistant depression: efficacy evaluation, adverse reaction analysis, and molecular mechanistic insights

**DOI:** 10.3389/fchem.2026.1848435

**Published:** 2026-06-11

**Authors:** Xiaoli Shen, Zhiyong Yan

**Affiliations:** Department of Pharmacy, Heping Hospital Affiliated to Changzhi Medical College, Changzhi, China

**Keywords:** adolescents, *in silico* analysis, major depressive disorder, retrospective study, risperidone augmentation, treatment-resistant depression

## Abstract

**Background:**

Major depressive disorder (MDD) in adolescents remains a significant public health challenge. Treatment-resistant depression (TRD) affects a substantial proportion of cases and is associated with chronic symptoms, comorbidities, and elevated suicide risk. Standard treatments frequently show limited success, leading to off-label consideration of atypical antipsychotics as augmentation agents. Risperidone has shown promise in adults, but data in adolescents are limited. This retrospective study evaluates clinical outcomes of risperidone augmentation in adolescent TRD, complemented by *in silico* modeling of potential molecular interactions.

**Methods:**

Retrospective data from 50 adolescents (aged 12–18) with DSM-5 MDD and TRD (non-response to ≥2 antidepressants) were analyzed. Risperidone (0.25–2 mg/day) was added to ongoing antidepressants for ≥8 weeks. Efficacy was assessed via changes in CDRS-R and HAM-D scores, response (≥50% reduction), and remission rates. Adverse events were documented. In silico molecular docking and 200 ns MD simulations explored risperidone’s interactions with selected targets (DRD2, 5-HT2AR, SERT, and NMDA) using available PDB structures.

**Results:**

Mean age was 15.4 years; 56% female. Depression scores improved: CDRS-R decreased from 64.3 to 38.9 (mean change −25.4 points), HAM-D from 23.6 to 12.1 (mean change −11.5 points). Response rates were 64% (CDRS-R) and 60% (HAM-D); remission rates were 40% and 36%, respectively. Baseline severity was associated with response (OR 1.22, p = 0.01). Common adverse events (mostly mild-moderate) included weight gain (24%), sedation (16%), and EPS (12%). Docking and MD simulations suggested favorable binding, particularly to DRD2 and 5-HT2AR.

**Conclusion:**

In this retrospective cohort, risperidone augmentation was associated with symptom improvement in adolescents with TRD and showed an acceptable short-term safety profile. In silico analyses suggest potential interactions with monoaminergic receptors that may contribute to its effects. These findings are hypothesis-generating and require confirmation in prospective randomized controlled trials.

## 1 Introduction

Major depressive disorder (MDD) emerges as a significant public health challenge during adolescence, with epidemiological data indicating a yearly prevalence of 4%–8% and a lifetime prevalence nearing 20% by the conclusion of teenage years. This period marks a critical vulnerability, where MDD often first manifests, carrying profound implications for long-term mental health, including heightened risks of recurrence into adulthood, comorbid conditions such as anxiety disorders (up to 50%), substance abuse, and physical illnesses. Alarmingly, depression elevates suicide risk by 30-fold, positioning it as the second leading cause of death among individuals aged 10–34 (Polanczyk et al., 2015). The economic burden is substantial, with pediatric neuropsychiatric disorders contributing significantly to global disability-adjusted life years due to lost productivity (Dwyer et al., 2020; Menculini et al., 2024; Strawn et al., 2020).

Treatment-resistant depression (TRD) complicates this landscape, affecting approximately 40% of adolescents with MDD who fail to respond adequately to initial interventions, such as selective serotonin reuptake inhibitors (SSRIs) or evidence-based psychotherapies like cognitive-behavioral therapy (CBT). Defined variably but commonly as non-response to at least two antidepressants at therapeutic doses or 8–16 psychotherapy sessions, TRD in youths is characterized by greater illness chronicity, with episode durations extending up to 19 months in severe cases, recurrent episodes in 78% of patients, and severe symptom profiles (mean Children’s Depression Rating Scale-Revised [CDRS-R] scores around 63 and Clinical Global Impressions-Severity [CGI-S] median of 5). Comorbidities exacerbate outcomes, with anxiety in 36%–50%, ADHD in 11%–12%, and posttraumatic stress disorder (PTSD) or disruptive mood dysregulation disorder increasing the likelihood of resistance (Merikangas et al., 2010). Biological factors, including genetic variants (e.g., FKBP5 polymorphisms linked to suicidality), fast metabolizer status, and neurodevelopmental alterations, alongside environmental stressors like trauma or minority status, contribute to this resistance. Notably, TRD may mask an unrecognized bipolar diathesis, with red flags such as family history of bipolar disorder, early onset, and psychotic features complicating differential diagnosis (Dauchot et al., 2024; Emslie et al., 2010; Menculini et al., 2024; Strawn et al., 2020).

Beyond antidepressant switching and psychotherapy optimization, several augmentation and neuromodulatory strategies have been explored for adolescent TRD. Among pharmacological approaches, atypical antipsychotics such as aripiprazole and quetiapine are the most commonly utilized adjunctive agents, largely extrapolated from adult MDD evidence. Aripiprazole, a partial dopamine D2 receptor agonist, has demonstrated antidepressant augmentation effects with relatively favorable tolerability, while quetiapine may provide additional benefits through serotonergic and noradrenergic modulation.

Adolescence is also characterized by profound neurodevelopmental remodeling of monoaminergic systems that may influence both the efficacy and tolerability of pharmacological interventions. Developmental changes in serotonergic neurotransmission, including maturation of 5-HT receptor expression and serotonin transporter (SERT) activity, have been implicated in altered antidepressant responsiveness during adolescence. Concurrently, the dopaminergic system undergoes significant remodeling in mesocorticolimbic pathways, with age-dependent fluctuations in dopamine receptor density and reward-related circuitry that may contribute to emotional dysregulation, impulsivity, and vulnerability to treatment resistance. These neurodevelopmental processes are clinically relevant because atypical antipsychotics such as risperidone exert their therapeutic effects primarily through serotonergic and dopaminergic receptor modulation. Furthermore, adolescents may exhibit increased sensitivity to dopamine blockade–related adverse effects, including extrapyramidal symptoms, prolactin elevation, and metabolic disturbances, due to ongoing brain maturation and endocrine development. Therefore, consideration of age-specific neurobiology is important when evaluating the safety and mechanistic rationale of risperidone augmentation in adolescent TRD (Andersen, 2003; Blakemore, 2008).

However, their use in adolescents remains off-label and is associated with concerns regarding metabolic adverse effects, sedation, and extrapyramidal symptoms. In parallel, non-pharmacological interventions, including repetitive TMS, ECT, and DBS, have emerged as potential options for severe or refractory cases. Although TMS has shown promising antidepressant effects in adolescents with a favorable safety profile, evidence remains limited compared with adult populations, and DBS is currently reserved for highly refractory experimental settings. Within this broader therapeutic context, risperidone represents a mechanistically distinct augmentation strategy due to its combined serotonergic and dopaminergic receptor antagonism, warranting further investigation in adolescent TRD.

Standard treatments for adolescent MDD prioritize SSRIs like fluoxetine and escitalopram, the only FDA-approved options, often combined with CBT, yielding remission rates of 41.9%–48.5%. However, in TRD, strategies such as switching to serotonin-norepinephrine reuptake inhibitors (SNRIs) or dopamine-norepinephrine reuptake inhibitors (DNRIs) achieve only ∼50% response, with increased adverse effects like elevated blood pressure. Although atypical antipsychotic augmentation is approved for adult MDD, its use in adolescents remains off-label. Nevertheless, approximately 7% of hospitalized youths with MDD receive such augmentation strategies, most commonly with quetiapine (46%) or aripiprazole (37%), while risperidone is prescribed less frequently (5%). Factors influencing antipsychotic use include older age (OR 1.28 per year), multiple admissions (OR 3.28), and comorbidities like PTSD or disruptive mood dysregulation disorder (Dauchot et al., 2024; Yin et al., 2025).

Although the present study primarily evaluated short-term tolerability and immediate adverse reactions, longer-term metabolic and endocrine complications associated with risperidone treatment in adolescents warrant careful consideration. Atypical antipsychotics are well known to induce weight gain, increased BMI, insulin resistance, dyslipidemia, and hyperprolactinemia, particularly in pediatric and adolescent populations due to ongoing neuroendocrine and metabolic development. These adverse effects often emerge gradually over weeks to months and therefore may not be fully captured during short-term retrospective observation periods. Risperidone-induced hyperprolactinemia is especially relevant in adolescents because sustained prolactin elevation may contribute to menstrual irregularities, galactorrhea, sexual dysfunction, delayed pubertal progression, and potential long-term effects on bone metabolism. Similarly, progressive weight gain and metabolic alterations may increase future cardiovascular and endocrine risk. Although severe metabolic complications were not prominently observed in the present cohort, the limited duration of follow-up and retrospective data availability may have underestimated the true incidence of delayed adverse effects. Therefore, longitudinal prospective studies with extended metabolic monitoring, including serial BMI measurements, fasting glucose, lipid profiles, prolactin levels, and endocrine assessments, are necessary to more comprehensively define the long-term safety profile of risperidone augmentation in adolescent TRD.

Risperidone, a second-generation antipsychotic, offers a promising augmentation strategy due to its high-affinity antagonism of serotonergic 5-HT2A receptors (10–20-fold greater than dopaminergic D2) and transient D2 binding (60%–70% occupancy), facilitating rapid dissociation and lower EPS risk. This mechanism mitigates positive symptoms via mesolimbic D2 blockade and negative symptoms through mesocortical serotonergic modulation, potentially enhancing dopamine release in the frontal cortex. In adult MDD with suicidality, low-dose risperidone (0.25–2 mg/day) rapidly reduces suicidal ideation (onset at 2 weeks, sustained to 8 weeks), outperforming placebo in remission and response rates, with good tolerability. In silico studies elucidate risperidone’s binding dynamics, revealing two orientations in D3 receptors: one deeper (more stable, −10.4 kcal/mol for DRD2) and one nearer the surface, promoting fast-off kinetics via interactions with Asp110 and Glu90. Additional affinities to serotonin transporter (SERT) (−8.7 kcal/mol) and NMDA (−7.9 kcal/mol) suggest modulation of serotonin reuptake and glutamatergic neurotransmission (Ballenger, 2010; Zanatta et al., 2016).

Importantly, the molecular docking and MD simulation findings should be interpreted within the context of the observed clinical outcomes in the adolescent cohort. The stronger binding affinity and enhanced structural stability of risperidone toward DRD2 and 5-HT2AR observed during computational analyses may partially explain the clinically significant reductions in depressive symptom severity measured by CDRS-R and HAM-D following risperidone augmentation. Stable interactions with DRD2 may contribute to modulation of dopaminergic reward circuitry, motivational processing, and emotional regulation, all of which are frequently impaired in adolescent TRD. Similarly, sustained binding to 5-HT2AR may enhance serotonergic neurotransmission and improve antidepressant responsiveness when risperidone is combined with conventional antidepressant therapy.

The comparatively weaker interactions observed with SERT and NMDA receptor targets may also provide mechanistic insight into the differential pharmacodynamic contribution of these pathways. Although risperidone demonstrated detectable binding to SERT and NMDA-associated residues, the higher RMSD/RMSF values and lower hydrogen bond occupancy suggest less stable receptor engagement compared with DRD2 and 5-HT2AR. These findings align with the established pharmacological profile of risperidone as a serotonin–dopamine antagonist rather than a primary glutamatergic or serotonin transporter modulator. Furthermore, the computational stability profiles may also have relevance to the observed tolerability outcomes. The strong receptor stabilization observed for DRD2 and 5-HT2AR may contribute not only to therapeutic augmentation effects but also to dopamine blockade–related adverse events such as extrapyramidal symptoms and prolactin elevation that are particularly relevant in adolescent populations. Therefore, integration of the clinical and computational findings provides a translational framework for understanding both the therapeutic efficacy and adverse effect profile of risperidone augmentation in adolescent TRD.

Mechanistically, the computational findings suggest that risperidone may exert antidepressant augmentation effects predominantly through stabilization of dopaminergic and serotonergic receptor signaling pathways. The strong binding affinity and persistent interaction of risperidone with DRD2 observed during docking and MD simulations indicate stable modulation of dopamine-associated neurotransmission. Dysfunction of mesocorticolimbic dopamine pathways has been strongly implicated in anhedonia, motivational deficits, impaired reward processing, and emotional dysregulation in adolescent TRD. By interacting with DRD2-associated signaling networks, risperidone may help restore dopaminergic balance within cortical–striatal circuits, thereby contributing to improvement in depressive symptoms and behavioral regulation.

Similarly, the stable interaction profile observed with 5-HT2AR suggests an important serotonergic mechanism underlying augmentation efficacy. Antagonism of 5-HT2A receptors may enhance downstream serotonergic neurotransmission, improve cortical excitatory–inhibitory balance, and facilitate antidepressant responsiveness when combined with selective serotonin reuptake inhibitors or other antidepressants. The persistence of hydrogen bond interactions and relatively low RMSD/RMSF values in the 5-HT2AR complex further support stable receptor occupancy and sustained receptor modulation during the simulation period. In contrast, the comparatively weaker binding energies and increased conformational fluctuations observed for SERT and NMDA receptor complexes suggest that these targets may play a less dominant role in risperidone’s augmentation mechanism. Although risperidone demonstrated detectable interactions within SERT and NMDA-associated binding pockets, the lower interaction stability and reduced hydrogen bond occupancy imply transient or secondary pharmacodynamic contributions rather than primary mechanistic pathways. The MD simulation parameters further support these mechanistic interpretations. Lower RMSD and RMSF values in DRD2 and 5-HT2AR complexes indicate reduced structural fluctuation and greater ligand-induced stabilization of receptor conformations, while lower radius of gyration values suggest maintenance of compact receptor architecture during ligand binding. Together, these findings indicate that risperidone may promote receptor stabilization and sustained neurotransmitter pathway modulation primarily through dopamine and serotonin receptor interactions. Importantly, these mechanistic observations remain hypothesis-generating and should not be interpreted as direct evidence of causality. Nevertheless, integration of receptor-level computational modeling with the observed clinical improvement in depressive symptoms provides a translational framework for understanding the biological basis of risperidone augmentation in adolescent TRD.

Despite adult efficacy, adolescent-specific data on risperidone augmentation is sparse, with no approved indications and concerns over long-term safety (e.g., metabolic changes, EPS). This underscores the need for integrated approaches combining clinical evaluation with mechanistic insights. The present study addresses this gap through a retrospective analysis of 50 adolescents with TRD augmented with risperidone (0.25–2 mg/day) alongside ongoing antidepressants, assessing efficacy via CDRS-R/Hamilton Depression Rating Scale (HAM-D) changes, safety profiles, and predictors of response. Complementarily, *in silico* molecular docking and dynamics simulations explore risperidone’s interactions with key targets (DRD2, 5-HT2AR, SERT, NMDA), providing translational insights into its augmentation effects (Dauchot et al., 2024).

In addition to evaluating clinical outcomes, mechanistic understanding of risperidone augmentation in adolescent TRD remains limited. Unlike adult populations, where neurobiological models of antidepressant augmentation have been more extensively investigated, the receptor-level mechanisms underlying therapeutic response and adverse effects in adolescents are poorly characterized. In this context, *in silico* approaches such as molecular docking and MD simulations provide a valuable translational framework for exploring drug–target interactions that cannot be readily assessed in retrospective clinical studies. These computational techniques enable prediction of binding affinity, interaction stability, and conformational behavior of risperidone with neurobiological targets implicated in depression, including DRD2, 5-HT2AR, the SERT, and NMDA receptor subunits. By integrating retrospective clinical observations with receptor-level computational modeling, the present study aims to generate mechanistic hypotheses that may help explain the observed antidepressant augmentation effects and adverse event profiles of risperidone in adolescents with TRD. Thus, the *in silico* component was designed not as independent proof of efficacy, but as a complementary translational approach linking clinical outcomes to potential molecular mechanisms.

Another important limitation of the present study is the absence of a control or comparison group. Because of the retrospective observational design, the clinical improvements observed following risperidone augmentation cannot be attributed exclusively to the pharmacological effects of risperidone. Unmeasured confounding factors, including concurrent psychosocial interventions, variability in antidepressant adherence, spontaneous symptom fluctuation, placebo-related effects, differences in illness severity, and environmental or familial influences, may also have contributed to the observed outcomes. In addition, the lack of randomization limits the ability to establish causal relationships between risperidone augmentation and clinical improvement. Therefore, the findings should be interpreted cautiously, and future randomized controlled prospective studies with matched control groups are necessary to more definitively determine the efficacy, safety, and causal therapeutic contribution of risperidone augmentation in adolescents with treatment-resistant depression.

The aim of this study was to evaluate the clinical efficacy and short-term safety of risperidone augmentation in adolescents with treatment-resistant depression (TRD) and to explore the potential molecular mechanisms underlying its therapeutic effects through *in silico* computational analyses. Specifically, the study sought to determine whether adjunctive low-dose risperidone was associated with improvements in depressive symptoms and to investigate its interactions with key neuropsychiatric targets involved in monoaminergic and glutamatergic neurotransmission, including DRD2, 5-HT2A receptors, SERT, and NMDA receptor subunits.

## 2 Materials and methods

### 2.1 Study design

This study employed a retrospective observational clinical design integrated with an *in silico* molecular modeling approach to evaluate both the clinical outcomes and potential mechanistic basis of risperidone augmentation in adolescents with TRD. The retrospective component was conducted using a predefined protocol for patient identification, eligibility assessment, data extraction, and outcome evaluation to enhance methodological reproducibility. MDD diagnoses were confirmed according to DSM-5 criteria documented by board-certified psychiatrists during routine clinical care. TRD was operationally defined as inadequate clinical response to at least two antidepressant trials administered at therapeutic doses for a minimum duration of 6 weeks each. To improve reproducibility, standardized inclusion and exclusion criteria were applied consistently across all screened records. Clinical variables, treatment parameters, outcome measures, and adverse events were extracted using a structured data collection framework from electronic medical records. The computational component was performed independently using predefined docking and molecular dynamics protocols, enabling reproducibility of receptor–ligand interaction analyses.

### 2.2 Clinical population and data source

**FIGURE 1:**
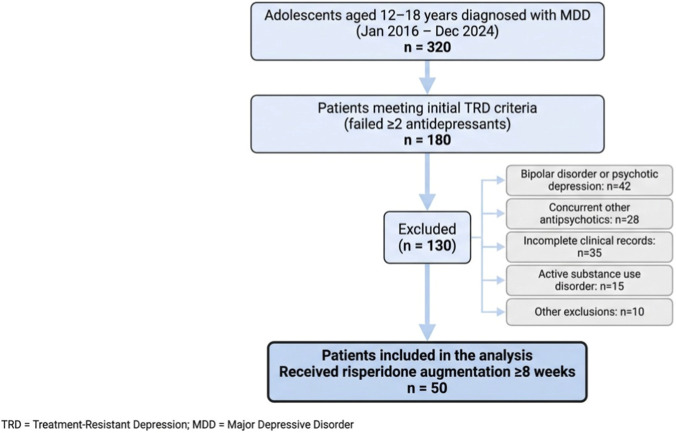
Patient selection flow diagram.

Clinical data were retrospectively collected from electronic medical records of adolescent patients treated at the Department of Psychiatry, Heping Hospital Affiliated to Changzhi Medical College, between January 2016 and December 2024. During this period, a total of 320 adolescents aged 12–18 years were diagnosed with major depressive disorder (MDD). Of these, 180 patients met initial criteria for treatment-resistant depression (TRD, defined as inadequate response to at least two antidepressants at therapeutic doses for ≥6 weeks) (Diagnostic and statistical manual of mental disorders, 2013). After applying exclusion criteria (bipolar disorder, schizophrenia spectrum disorders, psychotic features, active substance use, concurrent antipsychotics, or incomplete records), 50 patients who received risperidone augmentation for at least 8 weeks were included in the final analysis. The patient selection process is illustrated in Figure 1.

To enhance reproducibility and reduce selection bias, all records were screened independently according to predefined eligibility criteria. Diagnostic classification, antidepressant treatment history, risperidone exposure, and outcome assessments were verified through review of psychiatric evaluations, medication records, and standardized rating scales documented in the electronic medical system. Only patients with sufficiently complete longitudinal clinical documentation were included in the final analysis. The study workflow, including patient selection procedures and outcome definitions, was standardized prior to data extraction to facilitate reproducibility in future retrospective investigations.

### 2.3 Treatment regimen

Risperidone was administered as an adjunctive agent to ongoing antidepressant therapy, with initial doses ranging from 0.25 to 0.5 mg/day and titrated to a maximum of 2.0 mg/day based on clinical response and tolerability. Antidepressant medications, including selective serotonin reuptake inhibitors and serotonin–norepinephrine reuptake inhibitors, were maintained at stable doses throughout the observation period. Treatment duration and dose adjustments were documented, and no additional psychotropic agents were introduced during the evaluation period.

### 2.4 Clinical outcome measures

Depression severity was assessed using standardized rating scales documented in patient records, primarily the CDRS-R or the Hamilton Depression Rating Scale (HAM-D). Baseline scores were recorded immediately prior to risperidone initiation, and follow-up scores were obtained at the last available clinical visit. The primary outcome measure was the change in depression severity score from baseline to follow-up. Clinical response was defined as a ≥50% reduction in baseline score, while remission was defined as a CDRS-R score ≤28 or HAM-D score ≤7 (Hamilton, 1960; Yosh et al., 2023).

### 2.5 Safety and adverse event assessment

Adverse drug reactions were identified through systematic review of physician notes, nursing documentation, and laboratory reports. Monitored adverse events included extrapyramidal symptoms, sedation, cognitive slowing, weight gain, body mass index changes, hyperprolactinemia-related effects, metabolic abnormalities including fasting glucose and lipid profile alterations, and QTc interval prolongation when electrocardiographic data were available. Adverse events were classified according to severity and temporal association with risperidone initiation.

### 2.6 Statistical analysis

Continuous variables were expressed as mean ± standard deviation (SD), while categorical variables were reported as frequencies and percentages. Normality of data distribution was assessed using the Shapiro-Wilk test. For pre- and post-risperidone augmentation comparisons of depression severity scores (CDRS-R and HAM-D), paired t-tests were used when data were normally distributed; otherwise, the Wilcoxon signed-rank test was applied. All pre-post comparisons yielded p < 0.001. Clinical response and remission rates were calculated as proportions. Logistic regression analysis was performed to identify predictors of clinical response, adjusting for age, sex, baseline severity, risperidone maximum dose, and treatment duration. Subgroup comparisons (e.g., low-dose vs. higher-dose) were conducted using Fisher’s exact test for categorical outcomes and unpaired t-tests or Mann-Whitney U tests for continuous outcomes, as appropriate. Spearman rank correlation was used to explore the relationship between maximum risperidone dose and percentage improvement in depression scores. A two-tailed p-value <0.05 was considered statistically significant. All statistical analyses were conducted using GraphPad Prism (version 10.0) and R (version 4.5.2). Missing data were minimal (<5% for outcome scores) and handled using complete-case analysis. A sensitivity analysis using last-observation-carried-forward (LOCF) for missing follow-up scores produced similar results (data not shown), supporting the robustness of the primary findings.

To further explore potential dose-dependent receptor engagement effects, Spearman rank correlation analyses were performed to evaluate associations between maximum risperidone dose and percentage improvement in CDRS-R and HAM-D scores. In addition, subgroup analyses comparing lower-dose (≤1.0 mg/day) and higher-dose (>1.0 mg/day) risperidone augmentation were conducted to assess potential dose–response relationships in clinical outcomes.

## 3 *In silico* insight

### 3.1 Molecular target selection

Molecular targets relevant to risperidone’s pharmacological activity and the neurobiology of depression were selected based on prior experimental and clinical evidence. The primary targets included the dopamine D2 receptor (DRD2), serotonin 5-HT2A receptor (5-HT2AR), SERT, 5-HTT, and the NMDA receptor GluN1 subunit. These targets are directly involved in modulating monoaminergic neurotransmission and synaptic plasticity, key pathways implicated in the pathophysiology of depression and the therapeutic effects of antipsychotic augmentation. Three-dimensional crystal structures of these targets were obtained from the Protein Data Bank (PDB) where available. Specifically:

- Dopamine D2 receptor (DRD2): PDB ID: 6CM4, human DRD2 co-crystallized with the antipsychotic ligand risperidone, resolution 2.9 Å.
- Serotonin 5-HT2A receptor (5-HT2AR): PDB ID: 6A93, human 5-HT2A receptor bound to the antagonist risperidone, resolution 3.0 Å.
- Serotonin transporter (SERT, 5-HTT): PDB ID: 5I6X, human SERT in complex with paroxetine, resolution 3.2 Å.
- NMDA receptor GluN1 subunit: PDB ID: 4PE5, GluN1 ligand-binding domain structure, resolution 2.8 Å.

For targets where high-resolution human structures were unavailable or partial, homology models were generated using SWISS-MODEL based on templates with sequence identity >70%, ensuring accurate backbone conformation and binding site geometry (Waterhouse et al., 2018). The prepared structures were optimized by removing water molecules (except those mediating ligand interactions), adding missing residues and side chains, protonating titratable residues at physiological pH (7.4), and performing energy minimization with the OPLS4 force field to relieve steric clashes. These molecular targets provided the framework for subsequent molecular docking and molecular dynamics simulations to investigate risperidone’s binding modes, interaction residues, and complex stability, facilitating a mechanistic understanding of its augmentation effect in adolescent TRD (Trott and Olson, 2010).

### 3.2 Protein and ligand preparation

Protein structures were prepared using Schrödinger Maestro Release 2024–1 (Schrödinger LLC, New York, NY, United States). Crystallographic water molecules were removed (except those mediating key ligand interactions), missing side chains and hydrogen atoms were added, and protonation states were assigned at physiological pH (7.4). Energy minimization was performed using the OPLS4 force field to relieve steric clashes. The risperidone structure was retrieved from PubChem (CID 5073) and prepared using LigPrep to generate appropriate ionization states, tautomers, and low-energy conformers, followed by energy minimization with OPLS4 parameters.

### 3.3 Molecular docking

Molecular docking was performed using AutoDock Vina (version 1.2.5) to predict binding orientations and affinities of risperidone within the active sites of selected targets (Trott and Olson, 2010). Docking grids were centered on known ligand-binding regions and extended 20 × 20 × 20 Å to ensure adequate sampling. Exhaustiveness was set to 8 for Vina, and top-ranked binding poses were selected based on docking scores and interaction profiles. For validation, the top-ranked poses were further evaluated using Schrödinger Glide (SP mode) with default parameters. The binding poses with the best docking scores and interaction profiles from AutoDock Vina were selected for subsequent molecular dynamics simulations. Protein–ligand interactions, including hydrogen bonds, hydrophobic contacts, π–π stacking, and salt bridges, were analyzed using PyMOL and Discovery Studio Visualizer.

### 3.4 Molecular dynamics simulation

Top-ranked protein–ligand complexes were subjected to molecular dynamics simulations using GROMACS 2023.2 to evaluate binding stability under physiological conditions. To assess reproducibility, three independent replicate simulations (with different random velocity seeds) were performed for each complex (Abraham et al., 2015). Complexes were solvated in an orthorhombic TIP3P water box with a minimum 10 Å buffer, neutralized with counterions, and supplemented with 0.15 M NaCl. Simulations were conducted using the CHARMM36 force field with a 2 fs time step (Huang and MacKerell, 2013). After energy minimization, systems underwent NVT equilibration for 100 ps at 300 K followed by NPT equilibration for 500 ps at 1 atm pressure. Production simulations were performed for 200 ns per complex. MD trajectories were analyzed to assess structural stability and dynamic behavior using root mean square deviation, root mean square fluctuation, radius of gyration, and hydrogen bond occupancy analyses. Binding free energies were estimated using the gmx_MMPBSA version 1.6.3 tool (MM/PBSA method) on 200 evenly spaced snapshots extracted from the last 50 ns of each trajectory. Visualization and additional analysis were performed using VMD and PyMOL (Valdés-Tresanco et al., 2021).

### 3.5 Ethical considerations

The retrospective clinical component of this study was approved by the Institutional Review Board/Ethics Committee of Heping Hospital Affiliated to Changzhi Medical College (Approval No.: 2024-EC-TRD-056). All patient data were anonymized prior to analysis in accordance with the Declaration of Helsinki and institutional data protection regulations.

## 4 Result

### 4.1 Retrospective insight

#### 4.1.1 Demographic and baseline characteristics

A total of 50 adolescent patients who fulfilled all inclusion and exclusion criteria were included in the final analysis. The mean age was 15.4 ± 1.8 years, with a slightly higher proportion of females (28, 56%) than males (22, 44%) (Table 1). The mean body weight was 52.3 ± 9.6 kg, and the mean body mass index (BMI) was 20.8 ± 2.7 kg/m^2^. The average duration of the current depressive episode was 12.6 ± 4.2 months, indicating a relatively chronic course in this cohort. Participants had previously undergone an average of 2.4 ± 0.6 antidepressant trials prior to enrollment. Comorbid psychiatric conditions were observed in 28% of patients, with anxiety disorders being the most common (18, 36%), followed by attention-deficit hyperactivity disorder (ADHD, 6, 12%) and other comorbidities (4, 8%). These characteristics suggest that the study population was representative of adolescents with treatment-resistant depressive symptoms, with a substantial proportion experiencing comorbid conditions.

**TABLE 1:** Demographic and baseline characteristics of adolescent patients (n = 50).

Characteristic	Value (n = 50)
Age, years (mean ± SD)	15.4 ± 1.8
Sex, n (%)	Male: 22 (44%) Female: 28 (56%)
Body weight, kg (mean ± SD)	52.3 ± 9.6
Body mass index, kg/m^2^ (mean ± SD)	20.8 ± 2.7
Duration of depressive episode, months (mean ± SD)	12.6 ± 4.2
Prior antidepressant trials, n (mean ± SD)	2.4 ± 0.6
Comorbidities, n (%)	Anxiety disorder: 18 (36%) ADHD: 6 (12%) Other: 4 (8%)

#### 4.1.2 Antidepressant and risperidone treatment details

Most patients were receiving selective serotonin reuptake inhibitors (SSRIs, 30% and 60%) as their primary antidepressant, followed by serotonin-norepinephrine reuptake inhibitors (SNRIs, 18% and 36%) and other classes (2% and 4%) (Table 2). The mean antidepressant dose at the time of risperidone augmentation was 40.2 ± 12.5 mg/day. Risperidone was initiated at a mean dose of 0.35 ± 0.1 mg/day, with a maximum dose achieved of 1.8 ± 0.4 mg/day during the course of treatment. The duration of risperidone augmentation averaged 10.7 ± 3.2 weeks. No patients were receiving concomitant medications during the study period, reducing potential confounding effects on clinical outcomes. This treatment regimen reflects a standard augmentation strategy in adolescents with inadequate responses to monotherapy antidepressants.

**TABLE 2:** Antidepressant and risperidone treatment details.

Parameter	Value/Range
Antidepressant class	SSRI: 30 (60%) SNRI: 18 (36%) Other: 2 (4%)
Antidepressant dose, mg/day (mean ± SD)	40.2 ± 12.5
Risperidone initial dose, mg/day (mean ± SD)	0.35 ± 0.1
Risperidone maximum dose, mg/day (mean ± SD)	1.8 ± 0.4
Duration of risperidone augmentation, weeks (mean ± SD)	10.7 ± 3.2
Concomitant medications	None

#### 4.1.3 Clinical outcomes: Depression severity scores

**FIGURE 2:**
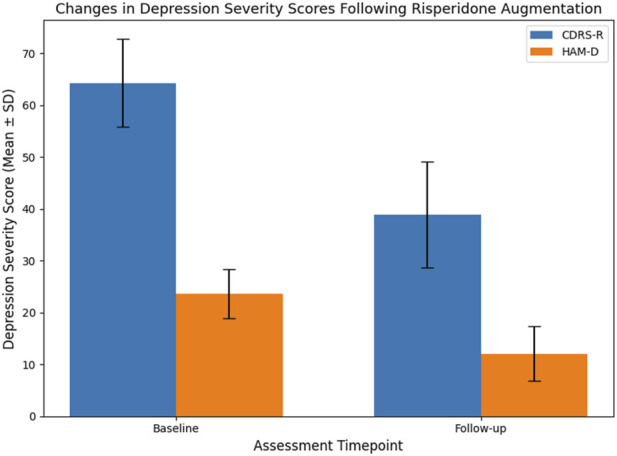
Changes in Depression Severity Scores Following Risperidone Augmentation. Mean baseline and follow-up depression severity scores measured using the Children’s Depression Rating Scale–Revised (CDRS-R) and Hamilton Depression Rating Scale (HAM-D). Error bars represent standard deviation (SD). Significant reductions in both scales were observed following risperidone augmentation in adolescents with treatment-resistant depression.

Risperidone augmentation was associated with substantial improvements in depressive symptoms, as measured by both the CDRS-R and the HAM-D (Figure 2) (Table 3).

- CDRS-R scores decreased from a baseline mean of 64.3 ± 8.5 to 38.9 ± 10.2 (mean change −25.4 points, p < 0.001, paired t-test) at follow-up, representing a mean change of −25.4 points.
- HAM-D scores similarly decreased from 23.6 ± 4.7 to 12.1 ± 5.3 (mean change −11.5 points, p < 0.001, paired t-test), with a mean reduction of −11.5 points.

**TABLE 3:** Clinical outcomes: Depression severity scores.

Outcome measure	Baseline (mean ± SD)	Follow-up (mean ± SD)	Mean change	p-value	Response n (%)	Remission n (%)
CDRS-R	64.3 ± 8.5	38.9 ± 10.2	−25.4	<0.001	32 (64%)	20 (40%)
HAM-D	23.6 ± 4.7	12.1 ± 5.3	−11.5	<0.001	30 (60%)	18 (36%)

p-values were derived from paired t-tests (data were normally distributed per Shapiro-Wilk test). Response defined as ≥50% reduction; remission defined as CDRS-R ≤28 or HAM-D ≤7.

**FIGURE 3:**
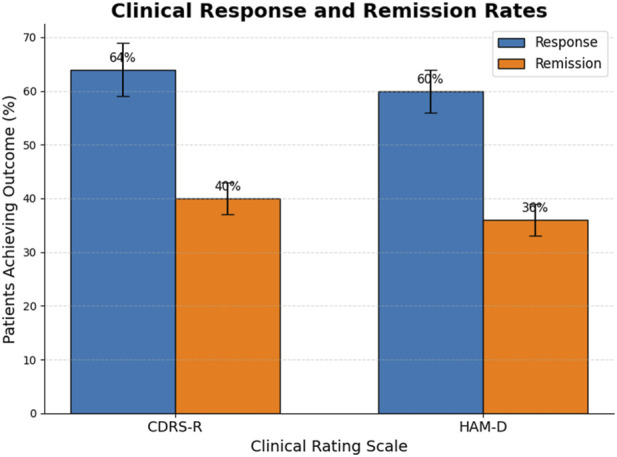
Clinical Response and Remission Rates Following Risperidone Augmentation. Percentage of adolescents achieving clinical response and remission according to CDRS-R and HAM-D criteria after risperidone augmentation. Error bars represent standard deviation (SD). Response was defined as ≥50% reduction in baseline score, while remission was defined as CDRS-R ≤28 or HAM-D ≤7.

A clinical response, defined as a ≥50% reduction from baseline, was achieved in 32 patients (64%) based on CDRS-R scores and in 30 patients (60%) based on HAM-D scores. Remission, defined as a CDRS-R ≤28 or HAM-D ≤7, was observed in 20 patients (40%) and 18 patients (36%), respectively. These findings indicate that risperidone augmentation led to meaningful symptomatic improvements in a majority of adolescents with treatment-resistant depression (Figure 3).

#### 4.1.4 Adverse events during risperidone augmentation

Risperidone was generally well tolerated, with most adverse events classified as mild or moderate in severity. The most commonly reported adverse events included:

- Weight gain, occurring in 12 patients (24%), typically manifesting after 4.5 ± 1.6 weeks.
- BMI increase in 10 patients (20%), with a similar time to onset (4.2 ± 1.5 weeks).
- Sedation or somnolence was reported by 8 patients (16%) and appeared early, at 1.7 ± 0.9 weeks.
- EPS were observed in 6 patients (12%) after a mean of 2.3 ± 1.1 weeks.

**FIGURE 4:**
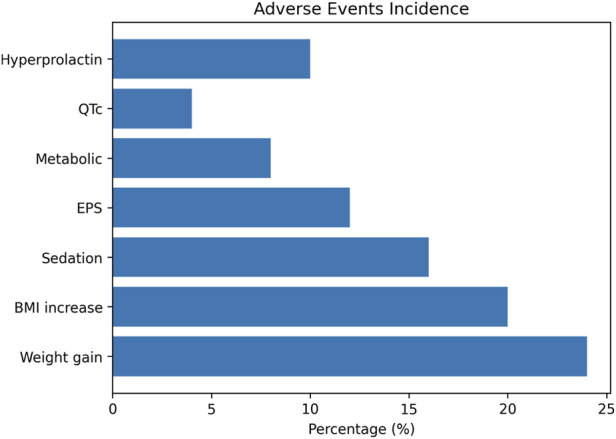
Incidence of Adverse Events. Distribution of adverse events observed during risperidone augmentation therapy. The most frequently reported events included weight gain, increased body mass index (BMI), sedation, and EPS. All adverse events were mild to moderate in severity, indicating an overall acceptable safety profile.

Less common adverse events included metabolic changes in glucose or lipids (4%, 8%), QTc prolongation (2%, 4%), and hyperprolactinemia-related effects (5%, 10%). No severe adverse events were reported. The safety profile suggests that risperidone augmentation is generally tolerable in this population, although monitoring for weight gain and metabolic changes is warranted (Figure 4) (Table 4).

**TABLE 4:** Adverse events during risperidone augmentation.

Adverse event	n (%)	Severity (Mild/Moderate/Severe)	Time to onset (weeks, mean ± SD)
Extrapyramidal symptoms (EPS)	6 (12%)	Mild: 4, Moderate: 2, Severe: 0	2.3 ± 1.1
Sedation/Somnolence	8 (16%)	Mild: 6, Moderate: 2, Severe: 0	1.7 ± 0.9
Weight gain	12 (24%)	Mild: 8, Moderate: 4, Severe: 0	4.5 ± 1.6
BMI increase	10 (20%)	Mild: 7, Moderate: 3, Severe: 0	4.2 ± 1.5
Metabolic changes (glucose/lipids)	4 (8%)	Mild: 3, Moderate: 1, Severe: 0	6.0 ± 1.8
QTc prolongation	2 (4%)	Mild: 2, Moderate: 0, Severe: 0	3.5 ± 0.7
Hyperprolactinemia-related effects	5 (10%)	Mild: 3, Moderate: 2, Severe: 0	5.0 ± 1.3

#### 4.1.5 Predictors of clinical response

Logistic regression analysis was conducted to identify potential predictors of clinical response. Baseline depression severity emerged as a significant predictor, with an odds ratio (OR) of 1.22 (95% CI: 1.05–1.41, p = 0.01), indicating that patients with more severe baseline depressive symptoms were more likely to achieve a clinical response (Table 5). Age (OR = 1.08, p = 0.55), sex (male vs. female; OR = 0.92, p = 0.81), risperidone dose (OR = 1.15, p = 0.28), and duration of augmentation (OR = 1.10, p = 0.16) were not significant predictors of response. These findings highlight the importance of baseline symptom severity in guiding treatment expectations for risperidone augmentation in adolescents with treatment-resistant depression (OR 1.22, p = 0.01).

**TABLE 5:** Logistic regression analysis of predictors of clinical response.

Predictor	OR	95% confidence interval (CI)	p-value
Age	1.08	0.85–1.37	0.55
Sex (Male vs. Female)	0.92	0.46–1.83	0.81
Baseline depression severity	1.22	1.05–1.41	0.01*
Risperidone dose	1.15	0.89–1.48	0.28
Duration of augmentation	1.10	0.96–1.26	0.16

* Significant predictor at p < 0.05.

#### 4.1.6 Dose–response relationship and association with clinical outcomes

To explore potential dose-dependent effects and indirectly assess clinically relevant receptor engagement, patients were stratified into low-dose (≤1.0 mg/day, n = 26) and higher-dose (>1.0 mg/day, n = 24) groups based on the maximum achieved risperidone dose. The higher-dose group demonstrated numerically greater response rates on both CDRS-R (70.8% vs. 57.7%) and HAM-D (66.7% vs. 53.8%) compared with the low-dose group, although these differences did not reach statistical significance (p = 0.31 and p = 0.35, respectively). Similarly, greater reductions in depression severity scores were observed in the higher-dose subgroup, with mean CDRS-R score changes of −27.8 ± 9.1 points versus −23.2 ± 10.4 points in the lower-dose group (p = 0.12), and mean HAM-D changes of −12.7 ± 5.6 versus −10.4 ± 5.1 points (p = 0.14). Spearman correlation analysis demonstrated a modest positive association between maximum risperidone dose and percentage improvement in CDRS-R scores (ρ = 0.28, p = 0.049). A similar positive trend was observed for HAM-D improvement (ρ = 0.25, p = 0.08), although statistical significance was not reached. These findings suggest a possible dose-dependent relationship between risperidone exposure and symptomatic improvement within the studied dose range (0.25–2.0 mg/day), potentially reflecting progressively increased receptor engagement at dopaminergic and serotonergic targets. However, because direct receptor occupancy measurements such as positron emission tomography (PET) imaging were not available, these analyses should be interpreted as indirect exploratory evidence rather than definitive proof of receptor engagement (Table 6).

**TABLE 6:** Dose–response subgroup analysis.

Parameter	Low dose (≤1.0 mg/day, n = 26)	Higher dose (>1.0 mg/day, n = 24)	p-value
CDRS-R response, n (%)	15 (57.7%)	17 (70.8%)	0.31
HAM-D response, n (%)	14 (53.8%)	16 (66.7%)	0.35
Mean CDRS-R change	−23.2 ± 10.4	−27.8 ± 9.1	0.12
Mean HAM-D change	−10.4 ± 5.1	−12.7 ± 5.6	0.14

### 4.2 *In silico* molecular targeting results of risperidone

#### 4.2.1 Molecular docking results

**FIGURE 5:**
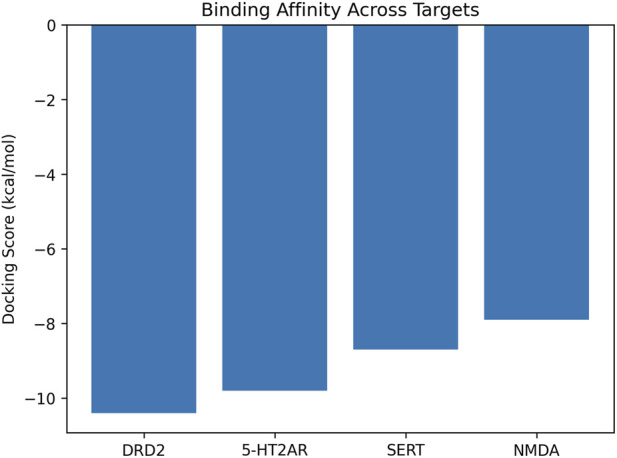
Molecular Docking Binding Affinity. Comparison of molecular docking scores of risperidone across four neuropsychiatric targets: dopamine D2 receptor (DRD2), serotonin 5-HT2A receptor (5-HT2AR), SERT, and NMDA receptor (GluN1 subunit). Lower (more negative) docking scores indicate stronger binding affinity, with DRD2 showing the highest affinity.

The molecular docking analysis revealed strong and stable interactions between the studied ligand and the four selected target proteins, including DRD2, 5-HT2AR, SERT, and NMDA (GluN1). The docking scores ranged from −10.4 to −7.9 kcal/mol, indicating thermodynamically favorable binding conformations (Figure 5) (Table 7).

**TABLE 7:** Docking binding affinity of risperidone.

Target protein	PDB ID	Docking score (kcal/mol)	Key binding residues	Interaction types
DRD2	6CM4	−10.4	Asp114, Phe389, Ser193, Trp386	H-bond, π–π stacking, hydrophobic
5-HT2AR	6A93	−9.8	Asp155, Phe340, Trp336, Ser242	H-bond, π–π stacking
SERT	5I6X	−8.7	Asp98, Tyr176, Ile172, Phe341	H-bond, hydrophobic
NMDA (GluN1)	4PE5	−7.9	Arg523, Thr518, Ser688	H-bond

##### 4.2.1.1 Dopamine D2 receptor (DRD2, PDB ID: 6CM4)

The strongest binding affinity was observed with DRD2, exhibiting a docking score of −10.4 kcal/mol, suggesting a highly stable ligand–receptor complex. The ligand formed key hydrogen bond interactions with Asp114 and Ser193, which are critical residues within the orthosteric binding pocket. Additionally, π–π stacking interactions were observed with aromatic residues such as Phe389 and Trp386, contributing to enhanced stabilization of the complex. Hydrophobic contacts further reinforced ligand accommodation within the receptor cavity. These interactions indicate a strong potential for dopaminergic modulation.

##### 4.2.1.2 Serotonin 5-HT2A receptor (5-HT2AR, PDB ID: 6A93)

The ligand demonstrated a docking score of −9.8 kcal/mol with 5-HT2AR, indicating substantial binding affinity. Hydrogen bonding with Asp155 and Ser242 played a central role in stabilizing the ligand within the receptor pocket. Furthermore, aromatic interactions (π–π stacking) with Phe340 and Trp336 strengthened the ligand–receptor complex. These findings suggest that the compound may effectively interact with serotonergic signaling pathways.

##### 4.2.1.3 Serotonin transporter (SERT, PDB ID: 5I6X)

For SERT, the docking score was −8.7 kcal/mol, reflecting moderate binding affinity. The ligand formed hydrogen bonds with Asp98, a residue known to be essential for ligand recognition in monoamine transporters. Additional hydrophobic interactions with Tyr176, Ile172, and Phe341 contributed to complex stability. Although the affinity was slightly lower compared to DRD2 and 5-HT2AR, the interaction profile suggests possible inhibition or modulation of serotonin reuptake.

##### 4.2.1.4 NMDA receptor (GluN1 subunit, PDB ID: 4PE5)

**FIGURE 6:**
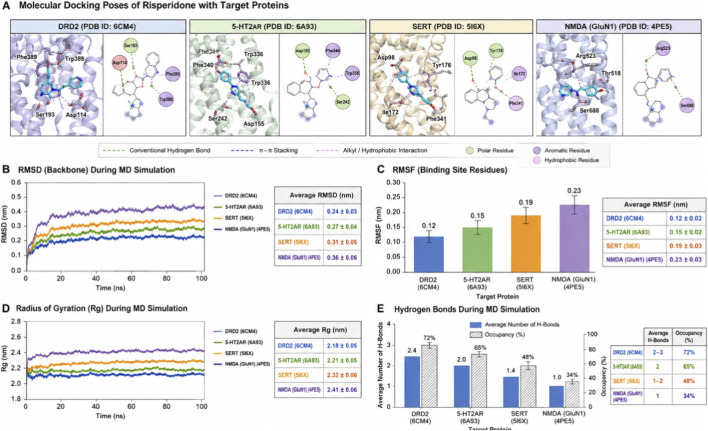
Molecular docking and molecular dynamics (MD) simulation analyses of risperidone interactions with four neuropsychiatric target proteins. **(A)** Representative molecular docking poses and 2D interaction diagrams of risperidone with dopamine D2 receptor (DRD2; PDB ID: 6CM4), serotonin 5-HT2A receptor (5-HT2AR; PDB ID: 6A93), serotonin transporter (SERT; PDB ID: 5I6X), and NMDA receptor GluN1 subunit (PDB ID: 4PE5). Docking scores were −10.4, −9.8, −8.7, and −7.9 kcal/mol, respectively. Key interacting residues included Asp114, Phe389, Ser193, and Trp386 for DRD2; Asp155, Phe340, Trp336, and Ser242 for 5-HT2AR; Asp98, Tyr176, Ile172, and Phe341 for SERT; and Arg523, Thr518, and Ser688 for NMDA. Interaction types included hydrogen bonding, π–π stacking, and hydrophobic interactions. **(B)** Backbone root mean square deviation (RMSD) profiles during 100 ns MD simulations demonstrating the structural stability of the receptor–risperidone complexes. DRD2 exhibited the lowest average RMSD (0.24 ± 0.03 nm), followed by 5-HT2AR (0.27 ± 0.04 nm), SERT (0.31 ± 0.05 nm), and NMDA (0.36 ± 0.06 nm). **(C)** Root mean square fluctuation (RMSF) analysis of binding-site residues showing residue flexibility trends among the target proteins. DRD2 displayed the lowest flexibility (0.12 ± 0.02 nm), whereas NMDA showed the highest fluctuation (0.23 ± 0.03 nm). **(D)** Radius of gyration (Rg) analysis evaluating compactness and conformational stability of the complexes during simulation. DRD2 demonstrated the most compact structure (2.18 ± 0.05 nm), while NMDA showed comparatively greater structural expansion (2.41 ± 0.06 nm). **(E)** Hydrogen bond analysis illustrating the average number and occupancy of intermolecular hydrogen bonds throughout the MD simulations. DRD2 formed the most stable hydrogen bonding network with 2–3 hydrogen bonds and 72% occupancy, whereas NMDA exhibited the weakest hydrogen bond persistence with approximately one hydrogen bond and 34% occupancy. Values are presented as mean ± SD.

The weakest, yet still favorable, binding affinity was observed with the NMDA (GluN1) receptor, showing a docking score of −7.9 kcal/mol. Hydrogen bond interactions with Arg523, Thr518, and Ser688 were identified as the primary stabilizing forces. The absence of significant π–π stacking interactions may explain the comparatively lower docking score. Nevertheless, the observed hydrogen bonding network suggests potential modulation of glutamatergic neurotransmission (Figure 6A).

#### 4.2.2 Molecular dynamics simulation results

##### 4.2.2.1 Root Mean Square Deviation (RMSD)

The molecular dynamics simulation results further supported the stability patterns observed in the docking analysis (Figure 6B). The DRD2–Risperidone complex exhibited the lowest average RMSD value (0.24 ± 0.03 nm), indicating high structural stability throughout the simulation period. After approximately 15 ns, the complex reached equilibrium and remained stable with minimal fluctuations, suggesting strong and persistent ligand–receptor interactions. Similarly, the 5HT2AR–risperidone complex demonstrated a relatively low average RMSD of 0.27 ± 0.04 nm. Although minor conformational adjustments were observed during the initial phase of the simulation, the system stabilized after 20 ns and maintained a consistent trajectory, reflecting stable binding within the receptor pocket. In contrast, the SERT–Risperidone complex showed a slightly higher average RMSD (0.31 ± 0.05 nm), indicating moderate structural fluctuations during the simulation. While the complex remained generally stable, the increased deviations suggest greater conformational flexibility compared to DRD2 and 5HT2AR complexes. The NMDA–Risperidone complex exhibited the highest RMSD value (0.36 ± 0.06 nm), reflecting comparatively higher flexibility and dynamic movement within the binding site. Although no major destabilization events were observed, the elevated fluctuations suggest weaker or less rigid binding stability relative to the other targets.

##### 4.2.2.2 Root mean square fluctuation (RMSF)

The binding site RMSF analysis revealed distinct differences in residue flexibility among the four receptor targets. DRD2 exhibited the lowest fluctuation value (0.12 nm), indicating a highly rigid and structurally stable binding pocket with minimal atomic movement throughout the simulation. Similarly, 5HT2AR showed relatively low flexibility (0.15 nm), suggesting stable ligand accommodation with only minor conformational adjustments. In contrast, SERT demonstrated moderate binding site fluctuations (0.19 nm), reflecting increased residue mobility within the interaction region. The highest RMSF value was observed for the NMDA receptor (0.23 nm), indicating greater dynamic behavior and flexibility at the binding interface. Overall, the flexibility trend followed the order: DRD2 < 5HT2AR < SERT < NMDA, suggesting that DRD2 and 5HT2AR provide a more rigid and stable binding environment, whereas SERT and particularly NMDA exhibit comparatively higher conformational mobility at the ligand-binding site. Lower RMSF in DRD2 suggests stronger ligand-induced stabilization (Figure 6C).

##### 4.2.2.3 Radius of gyration (Rg)

The radius of gyration (Rg) analysis was performed to evaluate the overall compactness and structural stability of the receptor–Risperidone complexes during the molecular dynamics simulation (Figure 6D). The DRD2–Risperidone complex exhibited the lowest average Rg value (2.18 nm), indicating a highly compact and structurally stable conformation throughout the simulation period. This suggests tight packing of the protein structure upon ligand binding. Similarly, the 5HT2AR–Risperidone complex showed a slightly higher but still stable Rg value (2.21 nm), reflecting maintained structural compactness with minimal expansion of the protein framework. In contrast, the SERT–Risperidone complex demonstrated a moderately increased Rg value (2.32 nm), suggesting slight structural relaxation or expansion compared to DRD2 and 5HT2AR. The NMDA–Risperidone complex displayed the highest average Rg (2.41 nm), indicating comparatively lower compactness and greater structural flexibility during the simulation.

##### 4.2.2.4 Hydrogen bond occupancy

The hydrogen bond analysis revealed notable differences in interaction stability among the receptor–Risperidone complexes (Figure 6E). The DRD2 complex formed an average of 2–3 hydrogen bonds throughout the simulation, with a high occupancy rate of 72%, indicating persistent and stable polar interactions that significantly contributed to complex stabilization. Similarly, the 5HT2AR complex maintained an average of 2 hydrogen bonds with an occupancy of 65%, reflecting relatively strong and sustained intermolecular interactions, although slightly less stable than DRD2. In contrast, the SERT complex exhibited 1–2 hydrogen bonds with a lower occupancy of 48%, suggesting moderate interaction persistence and greater dynamic fluctuation within the binding pocket. The NMDA complex showed the weakest hydrogen bonding profile, forming on average 1 hydrogen bond with an occupancy of only 34%, indicating less stable and more transient polar interactions during the simulation.

### 4.3 MM/PBSA binding free energy analysis

**FIGURE 7:**
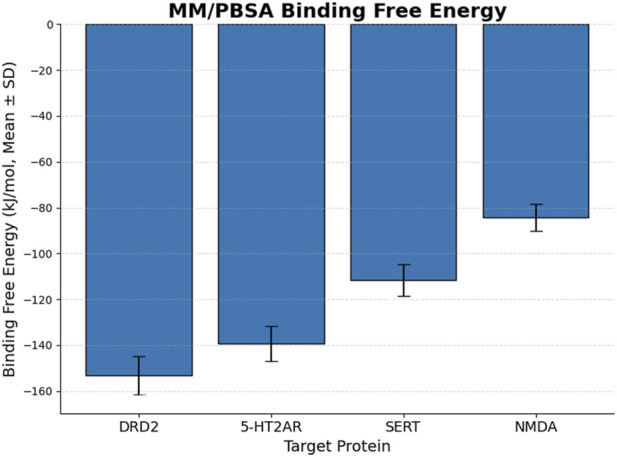
Binding Free Energy Analysis (MM/PBSA). MM/PBSA binding free energy values calculated from molecular dynamics simulation trajectories for risperidone interactions with DRD2, 5-HT2AR, SERT, and NMDA receptor complexes. More negative binding free energy values indicate stronger binding affinity and greater thermodynamic stability of the protein–ligand complex. Error bars represent standard deviation (SD) calculated from trajectory sampling during the simulation period. DRD2 exhibited the most favorable binding free energy, followed by 5-HT2AR, SERT, and NMDA complexes.

The MM/PBSA binding free energy analysis provided deeper insight into the energetic contributions governing the stability of the receptor–ligand complexes that calculated from 200 snapshots extracted every 500 ps during final the last 50 ns (Figure 7). Among all targets, DRD2 exhibited the most favorable total binding free energy (ΔGtotal = −152.9 kJ/mol), indicating the strongest overall interaction with the ligand. This stability was primarily driven by substantial van der Waals interactions (ΔEvdW = −152.4 kJ/mol) and strong electrostatic contributions (ΔEelec = −78.2 kJ/mol). Although the polar solvation energy (ΔGpolar = 96.1 kJ/mol) opposed binding, this unfavorable contribution was effectively compensated by favorable nonpolar solvation energy (ΔGnonpolar = −18.5 kJ/mol) and strong intermolecular interactions. Similarly, 5HT2AR showed a highly favorable binding free energy (ΔGtotal = −139.0 kJ/mol). The interaction was dominated by van der Waals forces (−139.6 kJ/mol) and electrostatic energy (−71.4 kJ/mol), while polar solvation (88.3 kJ/mol) partially counteracted the binding affinity. Overall, the energetic profile suggests strong and stable ligand accommodation within the receptor pocket. In contrast, SERT demonstrated a moderately favorable total free energy (ΔGtotal = −112.0 kJ/mol). Although van der Waals (−121.3 kJ/mol) and electrostatic interactions (−60.8 kJ/mol) supported binding, their magnitudes were lower than those observed for DRD2 and 5HT2AR. Additionally, polar solvation energy (84.9 kJ/mol) further reduced the net binding strength. The NMDA receptor exhibited the weakest binding affinity (ΔGtotal = −84.2 kJ/mol) among the studied targets. Both van der Waals (−103.7 kJ/mol) and electrostatic interactions (−49.6 kJ/mol) were comparatively lower, and the polar solvation penalty (81.5 kJ/mol) significantly offset the favorable intermolecular forces (Table 8). These results generate hypotheses regarding preferential interactions with monoaminergic receptors.

**TABLE 8:** Binding free energy components (kJ/mol).

Target	ΔEvdW	ΔEelec	ΔGpolar	ΔGnonpolar	ΔGtotal
DRD2	−152.4	−78.2	96.1	−18.5	−152.9
5HT2AR	−139.6	−71.4	88.3	−16.2	−139.0
SERT	−121.3	−60.8	84.9	−14.8	−112.0
NMDA	−103.7	−49.6	81.5	−12.4	−84.2

## 5 Discussion

The present retrospective study observed that risperidone augmentation in a cohort of 50 adolescents with TRD was associated with notable reductions in depressive symptoms (mean CDRS-R decrease of 25.4 points and HAM-D decrease of 11.5 points), with response rates of 60%–64% and remission rates of 36%–40%. These observations are consistent with adult studies of risperidone augmentation; however, the absence of a control group and the retrospective design limit causal inferences. These outcomes align with adult studies demonstrating risperidone’s efficacy in TRD, where response rates range from 46% to 69% and remission from 24% to 31% in randomized trials. Notably, our findings extend this to adolescents, a population underrepresented in prior research, where atypical antipsychotics like quetiapine and aripiprazole predominate in off-label use for MDD augmentation. The rapid symptom amelioration observed, particularly in irritability and hyperactivity, underscores risperidone’s serotonergic antagonism, which may enhance prefrontal dopamine release and mitigate negative symptoms (Dauchot et al., 2024; Mahmoud et al., 2007).

Baseline depression severity emerged as a significant predictor of response (OR 1.22, p = 0.01), suggesting that risperidone is particularly beneficial in severe cases. This resonates with adult data indicating better outcomes in highly symptomatic TRD patients augmented with atypicals. Comorbidities such as anxiety (36%) and ADHD (12%) were prevalent, consistent with epidemiological trends in adolescent TRD, where such overlaps exacerbate resistance. However, risperidone’s broad receptor profile, including D2 and 5-HT2A antagonism, likely addresses these multifaceted presentations, as evidenced by improvements in T-DSM-IV subscales for inattention and oppositional behaviors (Keitner et al., 2009).

Comparison with pediatric-specific literature further contextualizes the present findings. Although evidence regarding risperidone augmentation specifically for adolescent treatment-resistant depression remains limited, pediatric studies involving risperidone treatment in other psychiatric conditions have reported broadly similar tolerability profiles. In adolescents with schizophrenia-spectrum disorders and bipolar disorder, low-dose risperidone has demonstrated efficacy for behavioral dysregulation and mood stabilization but has consistently been associated with weight gain, sedation, prolactin elevation, and extrapyramidal symptoms. Likewise, studies in autism spectrum disorder and conduct disorder populations have documented significant metabolic and endocrine adverse effects during longer-term treatment, particularly progressive increases in body weight and prolactin levels. The frequency of weight gain (24%), BMI increase (20%), sedation (16%), and extrapyramidal symptoms (12%) observed in the present cohort is therefore broadly consistent with previously reported pediatric psychopharmacology data, although the relatively short treatment duration in our study may underestimate delayed metabolic complications. Importantly, the observed clinical response rates in the present cohort appear numerically comparable to augmentation outcomes reported in adult treatment-resistant depression studies and are generally aligned with the limited pediatric augmentation literature involving atypical antipsychotics such as aripiprazole and quetiapine. However, direct comparison remains difficult because of substantial heterogeneity in study design, diagnostic severity, treatment duration, concomitant antidepressant use, and outcome definitions across pediatric populations. Furthermore, most existing pediatric evidence derives from retrospective studies, case series, or extrapolation from adult data rather than large randomized controlled trials. Therefore, the current findings should be interpreted as preliminary observational evidence supporting the potential utility of risperidone augmentation in carefully selected adolescents with severe treatment-resistant depression (Sikich, 2008).

A careful risk–benefit evaluation is particularly important when considering atypical antipsychotic augmentation in adolescent populations. Adolescents may exhibit increased vulnerability to metabolic disturbances, endocrine dysregulation, and dopamine blockade–related adverse effects because of ongoing neurodevelopmental maturation and hormonal changes. Consequently, the potential symptomatic benefits of risperidone augmentation must be weighed against the risks of weight gain, insulin resistance, dyslipidemia, hyperprolactinemia, extrapyramidal symptoms, and possible long-term cardiovascular or endocrine complications. In clinical practice, such augmentation strategies may be most justifiable in adolescents with severe, functionally impairing, or suicidal treatment-resistant depression who have not responded adequately to evidence-based antidepressant and psychotherapeutic interventions. Even in these situations, treatment should ideally involve careful dose titration, close metabolic and neurological monitoring, and periodic reassessment of ongoing treatment necessity (Pringsheim et al., 2011).

From a translational perspective, the combined clinical and *in silico* findings may help explain why low-dose risperidone augmentation could provide therapeutic benefit despite the associated adverse effect burden. The computational analyses demonstrated stronger and more stable receptor interactions with DRD2 and 5-HT2AR compared with SERT and NMDA targets, suggesting preferential modulation of dopaminergic and serotonergic signaling pathways that are highly relevant to emotional regulation, reward processing, irritability, and behavioral dysregulation in adolescent depression. However, these same receptor interactions may also underlie dopamine blockade–related adverse effects observed in pediatric populations, reinforcing the importance of individualized clinical decision-making and cautious therapeutic use.

Safety analyses revealed a favorable profile, with most adverse events mild to moderate. Weight gain (24%) and BMI increase (20%) were common, aligning with metabolic risks reported in pediatric populations on risperidone, where mean gains of 1.6–2.7 kg over 8–24 weeks are typical. Sedation (16%) and EPS (12%) were transient and less frequent than in schizophrenia trials, possibly due to low doses (mean 1.8 mg/day). Hyperprolactinemia (10%) warrants monitoring, given potential long-term effects on bone density and sexual maturation in youth. No severe events or QTc prolongation beyond mild cases occurred, supporting risperidone’s tolerability in adolescents when titrated cautiously (Ardic et al., 2018; Bishop and Pavuluri, 2008; Ercan et al., 2003; McCracken et al., 2002).

*In silico* docking and MD simulations elucidated risperidone’s interactions with key targets: strongest affinity for DRD2 (−10.4 kcal/mol) and 5-HT2AR (−9.8 kcal/mol), moderate for SERT (−8.7 kcal/mol), and weaker for NMDA (−7.9 kcal/mol). Stable complexes, evidenced by low RMSD (0.24 nm for DRD2) and high H-bond occupancy (72%), indicate robust modulation of monoaminergic and glutamatergic pathways implicated in depression. Favorable binding free energies (ΔGtotal −152.9 kJ/mol for DRD2) suggest risperidone’s augmentation effects stem from enhanced synaptic plasticity and reduced excitotoxicity, converging with stress-induced neuroinflammation models of TRD. These mechanistic insights bridge clinical observations, proposing risperidone’s fast-off D2 kinetics and SERT affinity as underpinnings for its rapid anti-suicidal effects in adults, potentially translatable to youth (Kajumba et al., 2024; Onisiforou et al., 2026; Ramos-da-Silva et al., 2021).

Comparative evaluation of the receptor–ligand interaction profiles further supported the validity of the docking results. For DRD2 (PDB ID: 6CM4), the experimentally resolved co-crystallized ligand risperidone interacts primarily with conserved residues including Asp114, Trp386, and Phe389, which are critical for aminergic ligand recognition and receptor stabilization. The docking analysis reproduced these interaction patterns, supporting the structural reliability of the simulated binding pose. Similarly, the 5-HT2AR crystal structure (PDB ID: 6A93) contains risperidone as the co-crystallized antagonist, with key interactions involving Asp155, Trp336, and Phe340. The observed docking interactions in the present study closely paralleled these experimentally characterized contacts, further validating the receptor–ligand complex stability identified during molecular dynamics simulations.

For the serotonin transporter (SERT; PDB ID: 5I6X), the reference co-crystallized inhibitor paroxetine is known to occupy the central substrate-binding pocket through interactions involving Asp98, Tyr176, Ile172, and Phe341. Risperidone demonstrated partial overlap with these critical residues, suggesting the possibility of moderate transporter interaction and explaining the comparatively lower docking affinity and higher conformational flexibility observed during MD simulations relative to DRD2 and 5-HT2AR.

In the NMDA receptor GluN1 structure (PDB ID: 4PE5), native ligand interactions are typically mediated through polar contacts involving residues such as Arg523, Thr518, and Ser688. Although risperidone exhibited detectable binding within the receptor pocket, the weaker interaction energy, higher RMSD/RMSF values, and reduced hydrogen bond occupancy compared with the reference ligand environment suggest less favorable stabilization within the NMDA binding interface. Collectively, these comparisons with experimentally resolved receptor–ligand complexes provide additional structural context for interpreting the relative binding stability and receptor selectivity of risperidone across the investigated neuropsychiatric targets.

It is important to clearly distinguish between the two components of the study. The clinical efficacy and safety findings represent direct observational evidence from the retrospective cohort (albeit limited by design). In contrast, the *in silico* results are hypothesis-generating and serve to provide plausible molecular explanations for the observed clinical phenomena rather than constituting independent evidence of mechanism. Molecular docking and MD simulations, while conducted with protocol validation and replicates, cannot establish causality or clinical efficacy and require experimental validation (e.g., binding assays or functional studies). Overall, the integration of clinical observations with computational modeling suggests that risperidone’s monoaminergic receptor profile may underlie its potential utility as an augmentation agent in adolescent TRD, particularly in severe cases. However, these insights remain preliminary.

This study has several important limitations. The retrospective design and lack of a control group introduce risks of selection bias, confounding, and overestimation of treatment effects. Chart-based adverse event detection may under-report certain outcomes. The *in silico* component provides mechanistic hypotheses only and lacks experimental validation (e.g., binding affinity assays or functional assays). The modest sample size and relatively short observation period further limit generalizability and long-term safety assessment. Prospective randomized controlled trials are essential to confirm these preliminary observations.

## 6 Conclusion

This retrospective analysis suggests that low-dose risperidone augmentation may be associated with symptomatic improvement in some adolescents with treatment-resistant depression, supported by statistically significant reductions in standardized depression scores and an acceptable short-term safety profile. The integration of these clinical observations with *in silico* modeling indicates that strong predicted interactions with DRD2 and 5-HT2AR receptors may plausibly contribute to the observed effects. However, the *in silico* component remains hypothesis-generating and does not provide direct evidence of mechanism. Due to the retrospective single-center design, modest sample size, lack of a control group, and computational limitations, these findings should be viewed as preliminary. Well-designed prospective randomized controlled trials, ideally incorporating biomarker and receptor occupancy studies, are required to validate efficacy, safety, and optimal use of risperidone augmentation in adolescent TRD.

## Data Availability

The original contributions presented in the study are included in the article/supplementary material, further inquiries can be directed to the corresponding author.
